# Polycystic ovary syndrome and recurrent pregnancy loss, a review of literature

**DOI:** 10.3389/fendo.2023.1183060

**Published:** 2023-10-30

**Authors:** Rosa Wartena, Mushi Matjila

**Affiliations:** Department of Gynecology and Obstetrics, Groote Schuur Hospital, Cape Town, South Africa

**Keywords:** polycystic ovary syndrome, recurrent pregnancy loss, RPL, pcos, recurrent miscarriage, metformin

## Abstract

**Objective:**

PCOS is a syndrome of ovarian dysfunction associated with recurrent pregnancy loss. Several correlating factors have been investigated that influence the risk of pregnancy loss in PCOS. However, uncertainty remains about their contribution to pregnancy loss and prognosis. This review of literature aims to identify what is known and what requires further investigation on the relationship between PCOS and recurrent pregnancy loss, to guide future research and optimize medical guidance throughout pregnancy.

**Study design:**

a review of literature was performed on several search engines using the following terms; polycystic ovarian syndrome, PCOS, recurrent pregnancy loss, recurrent miscarriage, RPL, aborted fetus, abortus provocatus, miscarriage and habitual abortion.

**Results:**

37 articles were included; 3 systematic reviews, 1 meta-analysis, 2 randomized controlled trials, 6 prospective cohort studies, 22 case-control studies and 3 case series. The main objectives investigated by studies were pregnancy complications, pregnancy loss and live birth in the PCOS population.

**Conclusion:**

Studies that investigated the relationship between PCOS and recurrent pregnancy loss are few and inconsistent and warrant further research. Factors apt for further investigation include the extent to which PCOS phenotypes, BMI, obesity, insulin resistance, hyperandrogenemia, SHBG, hs-CRP, CTRP6, adiponectin, plasma leptin, homocysteine, AMH and thrombophilia contribute to further risk of miscarriage. Other factors requiring further exploration in relation to risk for miscarriage in PCOS patient with RPL include sOB-R, PAI-Fx and the Factor-V-Leiden mutations.

## 1 Introduction

Polycystic ovary syndrome (PCOS) is a syndrome of ovarian dysfunction and is currently diagnosed by the Rotterdam criteria. These criteria include 1. Oligo- or anovulation, 2. Clinical and/or biochemical signs of hyperandrogenism, 3. Polycystic ovaries on imaging (after exclusion of other etiologies). PCOS is diagnosed if two or more of these criteria are present (1). Clinical manifestations include signs of androgen excess such as hirsutism, acne or alopecia, menstrual irregularities, and obesity. Moreover, PCOS is associated with an increased risk of type 2 diabetes mellitus. Another clinical manifestation of PCOS is anovulatory infertility and thus women with PCOS may require assisted reproductive techniques (ART) to achieve pregnancy. Pregnancies in women with PCOS have been associated with more frequent pregnancy complications including recurrent pregnancy loss (2). An increased risk of Gestational Diabetes Mellitus (GDM), preeclampsia (PE), Pregnancy Induced Hypertension (PIH), preterm delivery, caesarean delivery, miscarriage, neonatal hypoglycemia, and perinatal death has been reported in the PCOS population (3). For women with PCOS it is critical to identify potential risk factors for miscarriage to potentially mitigate against further pregnancy loss. Results from studies performed on the relationship between PCOS and recurrent pregnancy loss are limited and often inconsistent. This review of literature intends to present an overview of known factors correlating PCOS and recurrent pregnancy loss, and further aims to identify biomarkers associated with this correlation to improve current pathophysiological understanding and potentially explore novel therapeutic interventions for PCOS associated pregnancy loss.

## 2 Methods

A search was performed on host search engines Pubmed, the Cochrane Library, ScienceDirect, Scopus and EBSCO using the following search terms; polycystic ovary syndrome, polycystic ovarian syndrome, PCOS, recurrent pregnancy loss, recurrent miscarriage, RPL, aborted fetus, abortus provocatus, miscarriage and habitual abortion. Filters used for study type: ‘Clinical Study’, ‘Clinical Trial’, ‘Clinical Trial Phase III’, ‘Clinical Trial Phase IV’, ‘Controlled Clinical Trial’, ‘Meta-Analysis’, ‘Multicenter Study’, ‘Randomized Controlled Trial’, ‘Systematic Review’, ‘Review articles’ and ‘Research Articles’. Filters used for Language: ‘Afrikaans’, ‘Dutch’, ‘English’. Filters used for Species: ‘Humans’. Articles published between 2000 and 2021 were included. Evaluation of articles was performed following the PRISMA-principle.

## 3 Results

**Figure 1:**
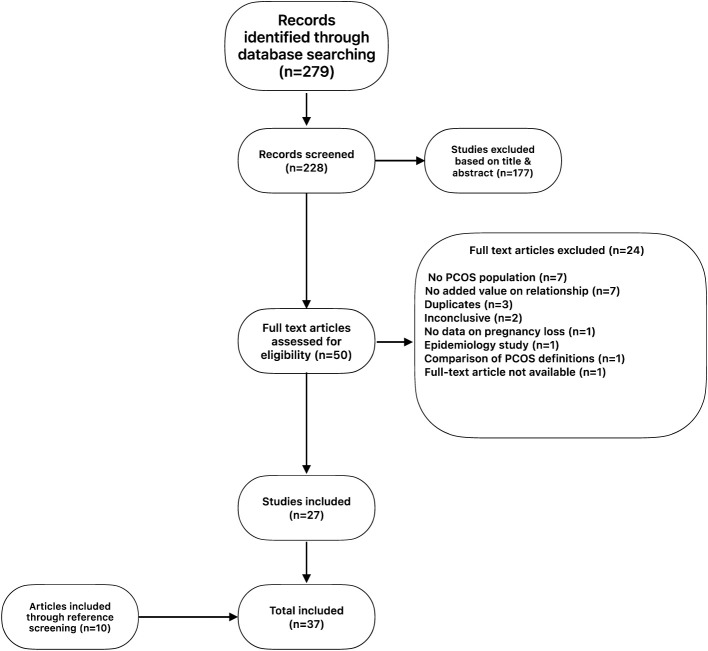
PRISMA flow diagram.

A total of 279 articles were found. Eligibility was established based upon title and abstract. Fifty (50) articles were selected with consideration of the inclusion and exclusion criteria (**Table 1**). After reading the complete articles, 27 articles were included for analysis. Additionally, by screening references, another 10 articles were retrieved. A total of 37 articles were included (**Supplementary Material**) and analyzed following the PRISMA principle (**Figure 1**). The complete search is available as supplement.

**Table 1:** in- and exclusion criteria.

Inclusion criteria	Exclusion criteria
Women with PCOS	Women without PCOS
	Women without RPL
(Recurrent) miscarriage or early recurrent pregnancy los	Focus of study does not match research question
Human subjects	Animal studies
Women of reproductive age	*In vitro* studies
English language	Cost-effectiveness studies
* *	Ovulation induction as study objective

Three systematic reviews, one meta-analysis, two randomized controlled trials, six prospective cohort studies, twenty-two case-control studies and three case series were included. Main objectives of the included studies were miscarriage rate and pregnancy complications which included gestational diabetes, pregnancy-induced hypertension, preeclampsia and coagulation disorders in the PCOS population. Additionally, studies that investigated live birth as an outcome mainly studied it in relation to metformin use in the PCOS population. Only two amongst these studies identified a PCOS-RPL population, highlighting an express need for the investigation of PCOS-associated RPL.

### 3.1 PCOS and pregnancy

#### 3.1.1 The role of BMI

A case-control study investigated endothelial dysfunction in non-pregnant PCOS women without identification of RPL (4) (**Table 2**). Levels of sex-hormone binding globulin (SHBG) were significantly lower and serum LH, testosterone, androstenedione levels, and LH to FSH ratio were significantly higher in the PCOS group when compared to non-PCOS controls. SHBG is a regulator of plasma levels and activity of sex hormones, and low SHBG levels generally correlate with high levels of bioavailable androgens (10). CRP levels were significantly increased in PCOS women. Even though CRP levels normally correlate with increased BMI, in this study, PCOS patients with normal BMI still demonstrated increased CRP levels. Other findings investigated across BMI categories were insulin resistance (IR) and hyperinsulinemia, which were reported in both lean and obese PCOS patients. A RCT investigating the effect of metformin on miscarriage and livebirths in obese and non-obese PCOS women with infertility found that obese women with PCOS were more insulin resistant, hyperinsulinaemic, hirsute and more hyperandrogenaemic than non-obese women (7).

**Table 2:** PCOS and pregnancy.

	*Main outcome*	*Reference*
*SHBG*	Significantly lower in PCOS group	Tarkun et al. (4)
*LH, testosterone, androstenedione, LH to FSH ratio*	Significantly higher in PCOS group Level of free testosterone associated with an increased risk of PCOS, PCOS-related infertility and RPL	Tarkun et al. (4) Kargasheh et al. (5)
*CRP*	Significantly increased in PCOS even with normal BMI	Tarkun et al. (4), Fouani et al. (6)
*BMI*	IR and hyperinsulinemia in both lean and obese PCOS patients Obese women with PCOS more insulin resistant, hyperinsulinaemic, hirsute and more hyperandrogenaemic than non-obese women Serum CTRP6 strongly associated with BMI	Morin-Papunen et al. (7) Morin-Papunen et al. (7) Sadeghi et al. (8)
*PCOS phenotypes*	Highest serum androgen levels: PCO + HA + AO phenotype	Ma et al. (2021)
*Plasma leptin & sOB-R*	Plasma leptin significantly higher and sOB-R lower in PCOS with infertility and RPL compared to non-PCOS controls Leptin levels elevate the risk of PCOS and the likelihood of RPL in PCOS	Kargasheh et al. (5) Kargasheh et al. (5)
*CTRP6*	Positively associated with fasting blood glucose, triglyceride levels and hs-CRP Negatively associated with adiponectin	Sadeghi et al. (8) Sadeghi et al. (8)
*Metformin*	Decreases fasting glucose levels, fasting insulin and serum total testosterone levels No effect on BMI, SHBG or serum lipids	Morley et al. (9) Morley et al. (9)

SHBG, Sex Hormone Binding Globulin.

IR, Insulin Resistance.

CTRP6, C1q/TNF- α-related protein 6.

PCO, polycystic ovarian morphology.

HA, hyperandrogenism.

AO, oligo-anovulation.

sOB-R, soluble leptin receptor.

#### 3.1.2 PCOS phenotypes

A meta-analysis was performed on pregnancy outcomes in patients with different PCOS phenotypes as these phenotypes could represent different pathophysiological entities (**Table 3**) (12). The highest serum androgen levels were found in PCOS women with polycystic ovary morphology + hyperandrogenism + oligo-anovulation. (i.e. the (PCO+HA+AO) phenotype).

**Table 3:** definitions of PCOS phenotypes.

*Study*	Phenotypes
*Ma et. al (2021)*	Hyperandrogenism - PCO + AO + HA - PCO + HA - AO + HA Normal androgen levels - PCO + AO
*Khomami et. al. (2019)* (11)	Ovulatory - HA + PCOM Anovulatory - AnOvu + HA - AnOvu + PCOM - AnOvu + HA + PCOM Hyperandrogenic - HA + PCOM - AnOvu + HA - AnOvu + HA + PCOM Non-hyperandrogenic - AnOvu + PCOM

#### 3.1.3 Proteins and PCOS entities

Plasma leptin and soluble leptin receptor (sOB-R) levels have been studied in PCOS patients in the context of RPL. A total of 324 PCOS patients were studied, which consisted of a 199 PCOS-infertile cohort and a 125 PCOS-RPL cohort. In addition, 144 healthy non-PCOS controls were included as comparator (5). In the PCOS group (with infertility and RPL), plasma leptin was significantly higher and sOB-R much lower in comparison to non-PCOS controls. A logistic regression analysis showed that raised leptin levels elevate the risk of PCOS and also the likelihood of RPL in PCOS women. The analysis additionally indicated that the level of free testosterone was associated with an increased risk of PCOS, PCOS-related infertility and RPL. Genotypic frequencies of leptin and leptin-receptor polymorphisms were also performed. No significant associations were observed between the genotypes of leptin (LEP (rs7799039)) and the leptin-receptor (LEP (rs1137101)) and clinical characteristics in PCOS-infertile and PCOS-RPL groups.

Another investigated protein was C1q/TNF- α-related protein 6 (CTRP6). CTRP6 is a newly identified adiponectin paralog. CTRP6 is expressed in multiple tissues including adipose tissue, heart, placenta and brain. It is proposed to be a regulator of multiple metabolic effects – mainly the regulation of complement activation (13). Moreover, higher levels of CTRP6 are associated with insulin resistance (14, 15). CTRP6 is moderately expressed in the ovarian follicle. The expression level of CTRP6 in follicular fluid gradually decreases along with the growth of the antral follicle. The concentration of CTRP6 in follicular fluid varies not only with the size of the follicle but CTRP6 expression is also stimulated by FSH, suggesting that it may be involved in FSH-modulated follicular development. Lastly, CTRP6 exists in the oocyte during follicular development, and may be associated with meiotic maturation of the oocyte and early embryonal development (16). Circulating serum levels of CTRP6 and adiponectin were studied in relation to inflammatory markers, sex hormones and metabolic variables in a PCOS-RPL group (n=60), a PCOS-infertile group (n=60) and a non-PCOS control group (n=60) (8). Triglyceride levels were significantly higher in the PCOS-RPL group. In both PCOS groups, levels of serum adiponectin were significantly lower and those of CTRP6 and hs-CRP were significantly higher in comparison to the control group. Serum CTRP6 levels were positively associated with fasting blood glucose, triglyceride levels and hs-CRP in the PCOS group and were strongly associated with BMI in all groups. Serum levels of CTRP6 were negatively associated with adiponectin in the PCOS group. These findings indicate a potential role for CTRP6 in the pathogenesis of PCOS but its importance for PCOS-RPL specifically remains unexplained.

#### 3.1.4 Metformin

Metformin had an effect of decreasing fasting glucose levels, fasting insulin and serum total testosterone levels in the PCOS population. The effect on testosterone was stronger in the non-obese subgroup who received metformin when compared to those who had placebo (9). In this study, metformin had no effect on BMI, SHBG or serum lipids (cholesterol or triglyceride levels) when compared to placebo.

### 3.2 Pregnancy Loss in PCOS and PCOS associated RPL

#### 3.2.1 The role of BMI and hyperandrogenism

Being overweight is a risk factor for miscarriage (11, 17). One study reported that high BMI and insulin resistance were associated with an increased risk of spontaneous abortion (SAB) in PCOS-RPL patients (18) (**Table 4**). This study showed higher HOMA-IR and BMI in the PCOS-RPL population when compared to the PCOS population who had successful pregnancies.

**Table 4:** Pregnancy Loss in PCOS and PCOS associated RPL.

	Main outcome	Reference
*BMI*	High BMI and IR associated with increased risk of SAB in PCOS-RPL	Asanidze et al. (18)
	Prevalence of miscarriage similar in women with and without PCOS with BMI >30kg/m^2^	Khomami et al. (11)
	Significant reduction in Metrnl in overweight/obese individuals	Fouani et al. (6)
*Hyperandrogenism*	Higher relative risk in miscarriage rate in PCOS with hyperandrogenism	Khomami et al. (11), Ma et al. (12)
*Androstenedione and testosterone levels*	Higher levels in RPL populations (with and without PCOS). Negative correlation between serum androgen concentrations and PP14	Okon et al. (19)
*PCO morphology*	PCO morphology without other Rotterdam Criteria was not predictive of an increased risk of pregnancy loss	Rai et al. (20)
*Metrnl*	Inverse correlation with markers of glucose homeostasis and inflammation in PCOS-RPL	Fouani et al. (6)
*Thrombophilia*	More thrombophilic disorders in PCOS-RPL (70,7%) vs non-PCOS RPL (47.8%)	Moini et al. (21)
	PAI-Fx independent variable for the number of miscarriages and worst pregnancy outcomes in PCOS. Correlation between the level of PAI-Fx and the rate of miscarriages	Glueck et al. (22)
	Higher levels of homocysteine in PCOS-RPL when compared to non-RPL PCOS	Asanidze et al. (18), Kazerooni et al. (23), Sadeghi et al. (8), Fouani et al. (6)
	Comparable levels of homocysteine between PCOS RPL and non-PCOS RPL	Moini et al. (21), Idali et al. (24)
	Higher prevalence of HHcy in PCOS-RPL vs non-PCOS RPL	Chakraborty et al. (25)
	HHcy negatively associated with pregnancy salvage	Chakraborty et al. (25)
*Insulin Resistance and Metformin*	Higher fasting blood glucose, fasting insulin and lower insulin sensitivity in PCOS-RPL vs. PCOS without RPL	Kazerooni et al. (23)
	Metformin decreased EPL (49.4% to 12%) in PCOS-RPL who conceived on and continued metformin vs. those who did not conceive on or discontinued metformin. Metformin reduces the risk of EPL from 53.8% to 11.1% in PCOS-RPL	Nawaz et al. (26), Jakubowicz et al. (27)
	Miscarriage rate decreased after metformin therapy in non-PCOS RPL as well as PCOS-RPL population when compared to placebo.	Zolghardi et al. (2008)
	In contrast, a RCT found no difference in early miscarriage rate between women who received metformin or placebo in a PCOS cohort without identification of RPL-subgroups	Morin-Papunen et al. (7)
	Two studies were inconclusive on the effect of metformin	Lovvik et al. (28), Morley et al. (9)
*AMH*	Higher AMH-levels in PCOS populations with miscarriages compared to PCOS with successful pregnancies. Chance of miscarriage increased by 90,8% for every 1ng/mL increase in AMH level when AMH ≥6,1 ng/mL.	Szafarowska et al. 29)
	No significant differences in serum AMH levels between PCOS with RPL and successful pregnancies	Asanidze et al. (18)

SAB, spontaneous abortion.

Metrnl, Meteorin-like protein.

PP14, placental protein 14.

PAI-Fx, Plasminogen Activator Inhibitor activity.

HHcy, hyperhomocysteinemia.

EPL, early pregnancy loss.

AMH, Anti-Mullerian Hormone.

Two systematic reviews observed a higher relative risk in miscarriage rate in PCOS women with hyperandrogenism. The first systematic review observed an increased miscarriage rate in the PCOS population independent of obesity, but the prevalence of miscarriage was similar in women with and without PCOS with BMI >30kg m^2^ – highlighting the importance of obesity over PCOS (11). The BMI of participants included in the other systematic review was comparable among all patients and also showed a higher miscarriage rate among PCOS patients with hyperandrogenism (12). Furthermore, the highest incidence of metabolic syndrome was observed in patients with the PCO + HA + AO phenotype. One prospective cohort study reported increased risk of spontaneous miscarriage in relation to high estradiol concentrations at the time of ovarian stimulation during Assisted Reproductive Technology (ART) (17). Another study observed higher circulating androstenedione and testosterone levels in RPL populations (with and without PCOS) and a negative correlation between serum androgen concentrations and those of uterine placental protein 14 (PP14). The PP14 levels were obtained through uterine flushings and endometrial biopsies (19). PP14 is also known as Glycodelin-A and has multiple functions in reproduction. PP14-levels peak during the first few weeks of gestation and contribute to immunosuppression, fertilization and implantation. A lower production of PP14 is associated with abnormal development of the endometrium and may affect implantation (30).

#### 3.2.2 PCO morphology

Prospective pregnancy outcomes in RPL+PCO women were compared to RPL women (20). The PCO population was identified as polycystic ovaries on imaging, without fulfilling the other Rotterdam criteria for the diagnosis of PCOS. The prevalence of PCO in 2199 women with RPL, was 40,7%. A total of 486 pregnancies with PCO phenotype were prospectively studied and showed that PCO morphology was not predictive of an increased risk of pregnancy loss.

#### 3.2.3 Meteorin-like protein

The adipokine Meteorin-like protein (Metrnl) was studied in the context of PCOS and RPL along with components of metabolic syndrome and cardiovascular biomarkers. It is involved in energy homeostasis and tissue inflammation and is suggested to increase insulin sensitivity (31–33). The study was performed in 180 women divided in three groups: PCOS-RPL (n=60), PCOS-infertile (n=60) and healthy controls (n=60) (6). In line with Sadeghi et. al., serum levels of hs-CRP were significantly higher in the PCOS-groups when compared to the control group and serum adiponectin and Metrnl levels were significantly lower. The Metrnl levels showed an inverse correlation with markers of glucose homeostasis and inflammation in patients diagnosed with PCOS and RPL, and a significant reduction in Metrnl levels was found in overweight/obese individuals in both PCOS groups when compared to healthy controls. The lowest levels of Metrnl were found in infertile obese PCOS patients. An independent association between serum levels of Metrnl and the diagnosis of PCOS was observed.

#### 3.2.4 Thrombophilia

Thrombophilic disorders were more frequently associated with PCOS than non-PCOS patients in a RPL population; a case-control study found that 70,7% of the PCOS-RPL versus 47.8% of the non-PCOS RPL population had associated thrombophilic disorders (21). Protein-C deficiency was significantly higher in the PCOS-RPL population when compared to the non-PCOS RPL population (21.7% and 10.9% respectively) and a non-significant trend toward a higher protein-S deficiency was additionally found. Three studies reported comparable Factor-V-Leiden mutation levels between PCOS and non-PCOS RPL groups (21, 34, 35) whereas one study found a higher prevalence of the mutation in the PCOS-RPL group (23). Glueck and colleagues performed multiple studies on coagulation and pregnancy loss (34–36). They identified high levels of Plasminogen Activator Inhibitor activity (PAI-Fx or PAI-I) as the major determinant of hypofibrinolysis, which is an important risk factor for miscarriage. PAI-Fx was an independent variable for the number of miscarriages and the worst pregnancy outcomes in PCOS patients. Moreover, PAI-I activity during the first trimester did not change or rose in PCOS women who had ≥1 previous SAB and/or their pregnancy ended in a first-trimester miscarriage whereas PAI-I activity lowered in the PCOS women with previous live births who had a successful pregnancy (22). The association between hypofibrinolysis and RPL in women with PCOS was subsequently confirmed by Kazerooni et al. and other workers (23). Glueck and colleagues concluded that first trimester miscarriages were not only associated with a higher mean PAI-Fx in PCOS women, but a correlation between the level of PAI-Fx and the rate of miscarriages was proposed. Moreover, multiple independent determinants of PAI-Fx activity were identified, including BMI, serum insulin levels and triglyceride levels (22).

Successful pregnancies are associated with lower levels of serum homocysteine and generally higher levels are associated with adverse pregnancy outcomes in a non-selected population (37). Four studies observed higher levels of homocysteine in the PCOS-RPL populations when compared to non-RPL PCOS populations [12,4 ± 1,6 vs. 7,3 ± 1,1 μmol L^-1^ (p<0,05) and 11,5 ± 2,24 vs. 7,55 ± 2,45 μmol L^-1^ (p<0,001)] (18, 23) and fertile controls [no exact data available and 13,29 ± 7,64 vs. 10,70 ± 3,69 μmol L^-1^ (p<0,05)] (6, 8). Furthermore, two studies reported comparable levels of homocysteine between PCOS and non-PCOS patients with RPL (21) (24).

A higher prevalence of hyperhomocysteinemia (HHcy) and insulin resistance was found in PCOS-RPL populations when compared to non-PCOS RPL populations (38). After controlling for IR, the correlation between the rate of miscarriage and HHcy became even greater. A prospective observational study, conducted partly by the same investigators, aimed to identify specific subgroups of RPL likely to benefit from the use of aspirin or a combination of aspirin and low molecular weight heparin (aspirin-LMWH) with the aim of improving pregnancy salvage (25). Aspirin was administered at a dose of 5MG/day and low molecular weight heparin at a dose of 2500 IU/day. The women in the cohort received aspirin for one conception cycle and if this conception cycle was unsuccessful, they received a combination of aspirin-LMWH for the second conception cycle. The total cohort consisted of 336 RPL patients (147 PCOS and 189 non-PCOS). The overall rate of pregnancy salvage in the total cohort was significantly lower in the PCOS population (32,65%) than in the non-PCOS population (51,32%). HHcy was negatively associated with pregnancy salvage. The selected cohort to receive Aspirin-LMWH consisted of 51,33% PCOS women. A trend towards higher pregnancy salvage rate was found in PCOS women treated with Aspirin-LMWH compared to non-PCOS women. However, the data show that aspirin-LMWH significantly increased pregnancy salvage rate in the RPL-HHcy phenotype that did not benefit from aspirin alone, irrespective of the presence or absence of PCOS, obesity or IR.

In a prospective cohort study, 19 PCOS women were compared before and after treatment with metformin-enoxaparin in terms of fetal loss rate. These women had 40 pregnancies without treatment and when they fell pregnant for a second time, they had 24 pregnancies with metformin-enoxaparin treatment (39). The fetal loss rate was 4.4-fold lower in the metformin-enoxaparin pregnancies. However, the sample size was small and no sub-analysis was performed on the number of previous miscarriages.

#### 3.2.5 Genotypic polymorphisms

The contribution of the M2 haplotype of the Annexin 5 gene (ANXA5) has been studied in PCOS related recurrent pregnancy loss (40). ANXA5 is involved in the prevention of coagulation at the maternal-fetal interface. Carriers of the M2 haplotype have decreased expression of ANXA5 in the placenta and peripheral blood, and manifest with RPL (41). A progressive correlation of M2 carriage with the number of miscarriages in the PCOS-RPL groups has been reported but there were no specific PCOS phenotypic characteristics that distinguished those with PCOS (without RPL) from patients with PCOS-associated RPL.

Frequencies of the methylenetetrahydrofolate reductase (MTHFR) A1298C polymorphism were significantly higher in the PCOS-RPL and PCO-RPL populations compared to healthy non-RPL controls (24). No significant differences in Activated Protein C Resistance (APCR), Protein C or Protein S deficiencies were found between RPL, RPL-PCOS and RPL-PCO populations. In line with Idali et. al., Szafarowska et al. found an increased miscarriage rate in non-PCOS RPL women with the A1298C polymorphism of the MTHFR gene (42).

Significant differences were observed in the IL-6-174G/C GG genotype frequency between PCOS-RPL and the control group (non-RPL non-PCOS population) (43). Significantly more GG-carriers were observed in the control group (65,2%) when compared to the PCOS-RPL group (60,0%).

#### 3.2.6 Insulin resistance and metformin

A prospective cohort study observed significantly higher levels of serum testosterone, dehydroepiandrosterone sulfate (DHEAS), fasting blood glucose, fasting insulin and lower insulin sensitivity in the PCOS-RPL population compared to the PCOS population without RPL (23).

Metformin is a relatively well-studied intervention in the context of PCOS and pregnancy loss (**Table 5**). Metformin decreased early pregnancy loss (EPL) (from 49.4% to 12%) in a cohort of women with PCOS - RPL who conceived on and continued metformin throughout the pregnancy in comparison to those who did not conceive on or discontinued metformin when pregnancy was confirmed (26). The study was performed in 197 PCOS patients of whom only 93 had a history of previous miscarriage. In the entire population (not solely RPL), the rate of early pregnancy loss was significantly decreased from 29.4% to 8.8% when metformin was continued throughout the pregnancy (n= 119) compared to when it was stopped (n=78). These findings are confirmed in a retrospective study which showed the risk of EPL in the metformin group (n=65) and the control group (n=31) was 8.8% and 41.9% respectively (27). In the PCOS-RPL cohort, the risk of EPL was reduced from 53.8% (control group, n=12) to 11.1% (metformin group, n=34). However, no stratification was performed on the number of previous miscarriages.

**Table 5:** timing and dose of metformin in relation to miscarriage rate.

	Timing of metformin	Dose	Miscarriage rate	Time of miscarriage
*Løvvik et al. (2019)*	From discovery of pregnancy until delivery	1000MG twice daily	Metformin group: 1%	Late miscarriage: between weeks 13 to 36 + 6 days
	- - Group 1: metformin (n = 244)		Placebo group: 2%	
	- - Group 2: placebo (n = 243)		p 0.72 (OR 0.60, 95% CI 0.09, 3.13)	
*Morin-Pupunen et al. (2012)*	Conceived on metformin, in case of pregnancy, continued up to the 12^th^ week of gestation	Obese women: 2000MG daily	Metformin group: 15.2%	Early pregnancy loss: first trimester
	- Group 1: metformin (n=160)	Non-obese women: 1500 MG daily	Placebo group: 17.8%	
	- Group 2: placebo (n=160)		p 0,70	
*Zolghadri et al. (2007)*	Conceived on metformin, continued throughout pregnancy.	1500MG daily	Group 1: 25%	Early pregnancy loss: first trimester
	- Group 1: PCOS + metformin		Group 2: 66%	
	- Group 2: PCOS + placebo		(p=0,42)	
	- Group 3: non-PCOS + metformin		Group 3: 15%	
	- Group 4: non-PCOS + placebo		Group 4: 55%	
			(OR 2.4, 95% CI 0.35-4.4, p=0,02)	
*Khattab et al. (2009)*	Metformin chronic therapy until conception	1000-2000MG daily (dose individualised)	Group 1: 11.6%	Early pregnancy loss: first trimester
	- Group 1: continuation during pregnancy (n = 120)		Group 2: 36.3%	
	- Group 2: discontinuation at time of conception or during pregnancy (n = 80)		p < 0,0001 (OR 0.23, 95% CI 0.11, 0.42)	
	
*Nawaz et al. (2009)*	Group 1: conceived on metformin, continued throughout pregnancy (n=119)	1500MG daily	Group 1: 8.8%	Early pregnancy loss: first trimester
	Group 2: discontinued metformin after conception or conceived spontaneously (n=78)		Group 2: 29.4%	
			p <0,001	
*Jakubowicz et al. (2002)*	Group 1: conceived on metformin and continued during pregnancy (n = 65)	1000-2000MG daily	Group 1: 8.8%	Early pregnancy loss: first trimester
	Group 2: did not receive metformin at time of conception or during pregnancy (n =31)		Group 2: 41.9%	
	* *		p < 0,0001	
*Glueck et al*. (44)	All women conceived on metformin	1500-2550MG daily	Group 1: 10.5%	Early pregnancy loss: first trimester
	- Group 1: continued throughout pregnancy (n = 19)		Group 2: 50%	
	- Group 2: stopped when pregnancy was detected at 4-6 weeks of gestation (n = 3)		statistical data not provided	
	
*Glueck et al*. (34)	Group 1: conceived on metformin, continued during pregnancy (n = 40)	1500-2250MG daily	Group 1: 26%	Early pregnancy loss: first trimester
	Group 2: same women & same partners. Metformin was stopped at end of first trimester (n = 40)		Group 2: 62%	
			p < 0,0001	
*Glueck et al*. (45)	Each woman is her own control	2000-2550MG daily	Group 1: 14%	Early pregnancy loss: first trimester
	- Group 1: conceived on metformin and continued during pregnancy (n = 76)		Group 2: 47%	
	- Group 2: same women but pregnancy without metformin (n = 76)		p 0,0004	
			(OR 3.99, 95% CI 1.91, 8.31)	

A number of studies were performed on the effect of metformin in the general non-RPL PCOS population. Five studies showed a reduction in miscarriage rate when metformin was administered (44–48). One study reported no difference in pregnancy outcomes between women who stopped metformin therapy after the first trimester, and those who continued metformin for the rest of their pregnancy (47). In a prospective cohort study, miscarriage rate was significantly decreased after metformin therapy in a non-PCOS RPL population when compared to placebo (15% and 55% respectively) (49). Metformin therapy was started before conception and continued throughout the pregnancy. The same study included a PCOS-RPL population which also showed a decreased miscarriage rate after metformin therapy when compared to placebo (25% vs. 66%) but failed to reach statistical significance. Of importance, study populations were small; the PCOS+metformin cohort consisted of 4 women, the PCOS+placebo cohort involved 3 women and the non-PCOS+metformin and non-PCOS+placebo cohort comprised of 13 and 9 women respectively. Furthermore, in the previously described studies, no sub-analysis on the history or number of previous miscarriages was performed. In contrast, a randomized, placebo-controlled study of 320 PCOS women, found no difference in early miscarriage rate between women who received metformin or placebo for three months pre-conception and, if conceived, continued metformin up to 12 weeks of gestation (7). However, in this study, no sub-analysis on the population with previous early miscarriages was performed, making it difficult to identify patients with RPL within the PCOS study cohort. Also, obese women received a higher dose of metformin than non-obese women; 2000mg/day and 1500mg/day respectively.

Two studies were inconclusive on the effect of metformin on pregnancy loss. One study investigated the effect of metformin (500mg twice daily) on late miscarriage (week 13 until week 22 + 6 days) in 470 women with PCOS (28). The study participants were randomized to receive metformin or placebo from detection of pregnancy until delivery. The study was underpowered and showed a non-significant reduction in late miscarriage: three (1%) and five (2%) late miscarriages occurred in the metformin and placebo groups respectively. A systematic review on the effect of metformin on miscarriage was inconclusive (9). No sub-analysis was conducted on recurrent miscarriages in both studies.

#### 3.2.7 Anti-Mullerian hormone

Anti-Mullerian hormone (AMH) has been studied as a predictive factor of pregnancy outcome in the PCOS population (29). A trend towards higher serum AMH levels was observed in the PCOS group (n=44) when compared to the non-PCOS control group (n=20), however PCOS populations that miscarried manifested significantly higher AMH levels than PCOS women who had a successful pregnancy. This contrasts with the findings by Asanidze et al. who reported no significant differences in serum AMH levels between PCOS patients with RPL and those with successful pregnancies. The study by Szafarowska et al. showed that the chance of miscarriage increased by 90,8% for every 1ng/mL increase in AMH level when AMH levels were ≥6,1 ng/mL (p=0,027) (29). All pregnancies in the studied population were following natural conception. The limiting factors for this study were the small sample size and that no sub-analysis was performed to distinguish PCOS patients with primary infertility from those with RPL.

### 3.3 Pregnancy morbidity amongst patients with PCOS and PCOS-associated RPL

Four studies (11, 12, 50, 51) note a relationship between PCOS and an increased chance of developing gestational diabetes (GDM), pregnancy induced hypertension (PIH), preeclampsia (PE), and premature delivery (**Table 6**). The risk of developing GDM and PIH or PE was independent of obesity in the studied groups.

**Table 6:** Pregnancy morbidity amongst patients with PCOS and PCOS associated RPL.

	Main outcome	Reference
*Pregnancy complications*	Increased chance of developing GDM, PIH, PE and premature delivery in PCOS group independent of obesity	Ma et al. (12), Khomami et al. (11), Boomsma et al. (50), Rees et al. (51)
*PCOS phenotypes*	Ovulatory, anovulatory and hyperandrogenic PCOS phenotypes: higher prevalence of GDM, gestational hypertension and PE independent of obesity. Prevalence of these outcomes similar in women with and without PCOS when BMI > 30kg/m^2^	Khomami et al. (11)
*Metformin*	Decreased rates of GDM, PIH, PE and IUGR	Nawaz et al. (26), Glueck et al. (47), de Leo et al. (46)
	Improved insulin sensitivity in PCOS women treated with metformin compared to no treatment.	Jakubowicz et al. (27)
	57% reduction in free testosterone levels in PCOS women with metformin compared to placebo	Jakubowicz et al. (27)

Two studies report a significantly higher chance of delivering prematurely in patients with PCOS in comparison to those without PCOS (50, 51).

#### 3.3.1 PCOS phenotypes

Khomami et al. reported a higher prevalence of GDM, gestational hypertension and PE for ovulatory, anovulatory and hyperandrogenic phenotypes of PCOS which was independent of obesity. The prevalence of these outcomes was similar in women with and without PCOS with BMI >30 kg/m2, which indicates a key role for obesity in the pathogenesis of these pregnancy disorders. However, no RPL population was identified within the studied PCOS population

#### 3.3.2 The effect of metformin on pregnancy complications

Aside from its effects on pregnancy loss, metformin was studied in the context of pregnancy complications. It significantly decreased the rates of gestational diabetes, pregnancy induced hypertension, preeclampsia and intrauterine growth restriction in the cohort that continued metformin throughout pregnancy (26). Moreover, Glueck et al. described a reduction in gestational diabetes from 26% to 4% for patients who continued metformin throughout their pregnancy (47). This reduction in GDM is likely mediated through decreased insulin resistance as Jacubowicz and co-workers showed improved insulin sensitivity in PCOS women treated with metformin (1000-2000mg daily) compared to those who did not receive treatment (27). Also, the PCOS women treated with metformin had 57% reduction in free testosterone levels compared to those who did not receive metformin. Additionally, de Leo et al. confirmed a reduction in GDM for those treated with metformin and showed a non-significant reduction in pregnancy hypertension and preeclampsia (46). In the untreated group (n= 110), 12 women developed GDM, 10 gestational hypertension and 3 preeclampsia whereas none in the metformin group (n=98) developed these complications. On the other hand, a study by Løvvik et al. showed no effect of metformin on the incidence of GDM or the need for insulin therapy, but demonstrated reduced weight gain for those on metformin. The study did report a non-significant reduction of preterm delivery (week 23 until week 36 + 6 days) for 9 (4%) cases in the metformin group compared to 18 (8%) in the placebo group. One study investigated the effect of metformin in combination with diet in pregnant women with PCOS (45). The diet comprised of 1500 or 1200 calories/day, was low in carbohydrate and fat and high in protein. Metformin + diet showed a reduction in preterm delivery (<37 weeks of gestation), GDM and preeclampsia.

### 3.4 Live birth

#### 3.4.1 PCOS phenotypes

The meta-analysis by Ma et al. not only studied miscarriage rate but also live birth rate (LBR) within different PCOS phenotypes. There was no statistically significant difference in LBR between the PCOS-hyperandrogenism and PCOS-normal androgen group (RR: 0.84, 95% CI: 0.69, 1.12), and there was no difference in LBR with further subgroup analysis of individual PCOS-phenotypes (**Table 7**). In the prospective cohort study by Rai et. al, which identified an RPL population with a PCO on imaging subgroup (not a PCOS subgroup), LBR were similar between the RPL group compared to the RPL-PCO group, and a non-significant increase in LBR was noted in those with elevated LH. LBR was similar between PCOS groups with elevated testosterone and normal testosterone as well as those with cycle lengths between >35 days and ≤35 days (20).

**Table 7:** Live Birth.

	Main outcome	Reference
*PCOS phenotypes*	No statistically significant difference in LBR between PCOS-HA and PCOS-normal androgen group and no difference in LBR with further subgroup analysis	Ma et al. (12)
	LBR Similar between RPL and RPL+PCO	Rai et al. (20)
	LBR similar between PCOS with elevated testosterone and normal testosterone as well as cycle lengths >35 days and ≤35 days	Rai et al. (20)
*Neonatal birthweight and perinatal morbidity and mortality*	Significantly lower birthweight of babies from PCOS mothers with no macrosomia. Babies from PCOS mothers more frequently admitted to the NICU and had significantly increased perinatal mortality compared to non-PCOS controls	Boomsma et al. (50)
	No difference in APGAR-scores in PCOS compared to non-PCOS but a significantly higher prevalence of jaundice and IRDS	Boomsma et al. (50), Rees et al. (51)
*Metformin*	LBR significantly increased in PCOS women treated with metformin compared to PCOS women without metformin	Nawaz et al. (26), Morin-Papunen et al. (7), Glueck et al. (47)
	Most beneficial effects of metformin in obese women	

LBR, Live Birth Rate.

IRDS, Infant Respiratory Distress Syndrome.

#### 3.4.2 Neonatal birthweight and perinatal morbidity and mortality

Boomsma et al. describes a significantly lower neonatal birthweight of babies born from PCOS mothers with no macrosomia compared to non-PCOS controls. This finding is unexpected as PCOS is usually associated with metabolic dysfunction, GDM and macrosomia. The finding of low neonatal weight may be a reflection of underlying placental dysfunction as the association between PCOS and hypertensive disorders of pregnancy is well described. Furthermore, babies from PCOS mothers were more frequently admitted to the neonatal intensive care unit (NICU) and had significantly increased perinatal mortality when compared to non-PCOS pregnancies. This may additionally be due to an increased premature delivery rate in the PCOS population since the reasons for NICU admission included hypoglycemia, jaundice and respiratory distress syndrome. Causes for perinatal death included lethal malformations, cervical insufficiency, sepsis and placental abruption. However, this study was conducted in a PCOS population with no identification of a RPL population. In contrast, Rees et al. did not report a difference in stillbirth nor Apgar scores in PCOS-complicated pregnancies compared to non-PCOS populations, but found a significantly higher prevalence of neonatal jaundice and respiratory distress syndrome and a trend toward lower birth weight and increased hypoglycemia in their PCOS population. Again, no RPL population was identified.

#### 3.4.3 The effect of metformin on live birth rates

LBR was also studied in relation to metformin use in the PCOS population. In the study by Nawaz et. al., LBR was significantly improved in PCOS women treated with metformin (500mg tds) (n=119) when compared to the PCOS population without metformin (n=78) - 92% and 70% respectively. The PCOS groups did not all manifest RPL, and no subgroup analysis was performed on the RPL populations. Likewise, the study by Morin-Papunen et al. showed a significantly higher LBR in the PCOS-metformin population when compared to the PCOS-placebo group. The most beneficial effects of metformin were found in obese women. The systematic review by Morin-Papunen and colleagues reported a modest effect of metformin on LBR in the PCOS population with a number needed to treat of 13 for one livebirth. A subgroup analysis examining BMI found no effect of BMI on LBR.

In the study by Glueck et al. favorable effects of metformin were found in the PCOS population (47). In this study, like many others, a RPL population was not identified. Seventy-two (72) women diagnosed with PCOS were treated with metformin (1500-2550MG/day pre-conception) and were compared to their own previous pregnancy outcomes without metformin treatment. On metformin there were 63 livebirths in 81 pregnancies (75%) whereas only 34 livebirths in 100 pregnancies (34%) were recorded in their previous pregnancies without metformin. In this study, pregnancy outcomes did not differ between women who continued metformin throughout the pregnancy versus women who discontinued it after the first trimester.

The previously described metformin+diet study which investigated 76 PCOS women showed an increase in live-births from 40 (53%) to 62 (82%) when metformin+diet was the intervention (45).

Lastly, Ramidi et al. studied enoxaparin-metformin combination in a PCOS population with ≥1 SAB (39). Dosage of metformin was 1500-2550MG/day and of enoxaparin 60MG/day. Nineteen women in 40 previous pregnancies had 7 live births (18%) without enoxaparin-metformin, and with enoxaparin-metfomin the same 19 women had 20 live births (83%) in 24 pregnancies. Twenty of these 24 births continued to term (91%).

## 4 Discussion

The intention of this review of literature was to present a clear overview of what is known about the relationship between PCOS and RPL, and which PCOS phenotypes might pose increased risk for further miscarriage or improved the chances of livebirth. Regrettably, studies that provided information on this relationship are scarce and results were frequently inconsistent. Given paucity of data, the review additionally included studies that explored potential mechanistic aspects behind PCOS associated pregnancy loss. The paucity of studies on data association between PCOS phenotypes and further risk of miscarriage and/or livebirth rates, particularly in PCOS patients with RPL, imposed a great a limitation of our study. Because results and studied groups were frequently inconsistent, this review portrays the need for clarification about the relationship and pathophysiology between PCOS and RPL.

The risk of GDM, PIH and PE was higher in the PCOS population without identification of a RPL population when compared to the non-PCOS population. A clear increase in spontaneous miscarriage risk was found in overweight PCOS patients. Being overweight and obese is a recognized risk factor for sporadic miscarriage. Also, miscarriage rate was frequently higher among PCOS patients with hyperandrogenism (although identification of PCOS patients with RPL was not conducted), however subgroup analyses of other PCOS phenotypes revealed no differences in miscarriage rate. Of importance, the definition of PCOS itself as well as that of its phenotypes varied widely between studies, making it difficult to compare study findings.

Multiple studies included PCOS patients undergoing ART, but no PCOS phenotypes or characteristics were identified and thus sub-analyses on the various techniques and the rate of miscarriage were not performed. This further raises the question of the potential influence of ART interventions on outcomes in PCOS populations.

Multiple studies reported elevated hs-CRP, triglycerides, insulin resistance and homocysteine and lower levels of adiponectin in the non-pregnant PCOS-RPL population. Women with PCOS and hyperinsulinemia have low-grade chronic inflammation which reflects the CRP levels and possibly causes endothelial dysfunction (48). Also, elevated levels of CTRP6 were found in the PCOS populations and were strongly associated with BMI. There might be a role for CTRP6 in the pathogenesis of PCOS but its contribution to PCOS-RPL remains unclear. The expression of Metrnl showed an inverse correlation with markers of glucose homeostasis and inflammation in the PCOS-RPL and PCOS-infertile population, however uncertainty remains on its contribution to RPL in PCOS since the pathogenesis of RPL might differ from that of PCOS with infertility.

Elevated levels of leptin and decreased levels of sOB-R were found in PCOS patients. Moreover, elevated leptin levels were shown to increase the risk of RPL in PCOS. However, no correlation between leptin polymorphisms and the serum levels of leptin were found. It therefore remains inconclusive whether elevated leptin levels drive the pathophysiology of RPL. Also, the level of free testosterone was associated with an increased risk of RPL in PCOS.

Information on metformin was occasionally contradictory and inconclusive. The most beneficial effects of metformin were found in obese women but, in some studies, the dose of metformin in obese women was higher than that used in non-obese women while in other studies identical doses were maintained. Additionally, some studies only included women with PCOS and IR in the PCOS population and compared this cohort to healthy controls with no PCOS or IR, raising the question of whether the effect of metformin was secondary to PCOS, IR or both. One study reported on effects of a combination of metformin and diet, but it became impossible to draw conclusions on the individual contributions of metformin or diet since both likely act synergistically.

Hypofibrinolysis was associated with non-pregnant PCOS-RPL women. Evidence on elevated levels of PAI-Fx in the non-pregnant PCOS population was coherent and associated with worse pregnancy outcomes in PCOS-RPL women. Also, in PCOS women with a history of ≥1 SAB, PAI-Fx levels remained unchanged or rose during the first trimester.

Higher levels of homocysteine and higher prevalence of hyperhomocysteinemia were found in the PCOS-RPL population. After controlling for IR, the correlation between the rate of miscarriage and HHcy became even greater. Pregnancy salvage was lower in HHcy irrespective of PCOS, IR or obesity which raises the contribution of HHcy in the PCOS-RPL population. Elevated AMH is a proposed biomarker for the risk of miscarriage in pregnant women with PCOS. Also, progressive correlation of M2 haplotype carriage with the number of miscarriages was found in the PCOS-RPL population but characteristics distinguishing the PCOS population from the PCOS-RPL population were not found. It therefore remains unclear whether M2 carriage is an independent risk factor of RPL irrespective of PCOS.

Babies from PCOS mothers were more frequently administered to the NICU and perinatal death was significantly higher in the PCOS population compared to the non-PCOS population which could be due to more frequent premature delivery or contribution from placenta mediated disease. Unfortunately, no RPL population was identified. Livebirth was mainly an outcome of studies that examined metformin therapy in the PCOS population. Overall, the studies show an increase in live birth and lower premature delivery rates in pregnant PCOS patients treated with metformin when compared to placebo or no treatment. This is important, as livebirth is the outcome of ultimate importance when PCOS affected couples are trying to conceive. Unfortunately, of the studies that reported on livebirth after metformin treatment, four studies partly included either RPL populations or populations that had ≥1 previous SAB’s but no sub-analyses on these sub-populations were performed and two studies did not identify the number of previous miscarriages.

Unfortunately, the underlying pathophysiological mechanisms behind PCOS associated RPL (e.g. inflammatory, metabolic, coagulation or most likely a combination of these), remains unclear.

Our study has some limitations. Firstly, the definition of RPL varied, with some studies defining recurrent pregnancy loss as ≥2 consecutive miscarriages whereas others used the definition of ≥3 consecutive pregnancy losses. Secondly, RPL populations were frequently included in the PCOS populations but were not specified for subgroup analysis. Often no data was available on the number of miscarriages per women and sub-analyses on this data were not performed. It therefore remains challenging to draw conclusions on outcomes when minimal information is available on the number of previous miscarriages in the studies, particularly when the RPL population with PCOS is not specifically identified. This is crucial as the risk of spontaneous abortion increases as the number of previous miscarriages is higher (52). Similarly, the chance of achieving livebirth will be lower as the number of previous miscarriages increases. Thirdly, several studies included only small sample sizes with no sample size calculations. The sample sizes became even smaller after subgroups were identified, further limiting the reliability of study findings. Also, the majority of studies applied the Rotterdam criteria to identify the PCOS but some studies used other criteria to define PCOS. Lastly, studies that define clinical phenotypes of PCOS in relation to pregnancy outcomes or RPL are scarce.

We propose studies that identify different PCOS phenotypes based on the previously described biochemical and clinical characteristics to differentiate PCOS patients at high risk for further pregnancy loss from those who will achieve successful pregnancies. This is necessary to optimize our understanding of PCOS-associated RPL, and in the future adapt medical guidance throughout pregnancy, as well as unravel novel therapeutic interventions aimed at optimizing livebirths and reducing the risk of pregnancy loss in pregnant women with PCOS.

## 5 Conclusion

We aimed to identify and characterize phenotypes that associate with increased risk of miscarriage in PCOS -associated RPL. Published studies on this subject are scarce and results are often inconsistent and confounded.

The extent to which BMI, obesity and PCOS phenotypes contribute to the risk of miscarriage in the PCOS populations with RPL as well as putative contributors to underlying pathophysiology, including, inter alia, insulin resistance, serum androgen levels, SHBG, hs-CRP, CTRP6, adiponectin, plasma leptin, homocysteine, AMH and thrombophilia warrant urgent exploration. Other elements include sOB-R, PAI-Fx and the Factor-V-Leiden mutations. Studies investigating this, along with those examining the relationship between PCOS-related RPL and other pregnancy morbidities such as gestational diabetes, preterm birth and hypertensive disorders of pregnancy will be welcome contributions to the field.

## Data availability statement

The original contributions presented in the study are included in the article/**Supplementary Material**. Further inquiries can be directed to the corresponding author.

## Author contributions

The authors confirm contributions to the paper as follows: study conception and design: RW, MM; Data collection: RW. Analysis and interpretation of results: RW. Draft manuscript preparation: RW. All authors contributed to the article and approved the submitted version.
