# Supplementary material for: Polycystic ovary syndrome and recurrent pregnancy loss, a review of literature
Source: Front Endocrinol (Lausanne). 2023 Oct 30;14:1183060. doi: 10.3389/fendo.2023.1183060 (PMC10643146; doi:10.3389/fendo.2023.1183060)
Supplement: Supplementary file 1 [file Table_1.docx]

Supplementary Material

Polycystic ovary syndrome and recurrent pregnancy loss, a reappraisal of literature

Rosa Wartena*, Mushi Matjila

*** Correspondence:** Rosa Wartena: rosawartena@hotmail.com

# Supplementary Data: search strategy

Pubmed:

((("polycystic"[All Fields] AND "ovarian"[All Fields] AND "syndrome"[All Fields]) OR ("polycystic"[All Fields] AND "ovary"[All Fields] AND "syndrome"[All Fields]) OR ("polycystic ovary syndrome" [All Fields]) OR PCOS [All Fields]) AND (("recurrent"[All Fields] AND "pregnancy"[All Fields] AND "loss"[All Fields]) OR ("recurrent miscarriage" [All Fields]) OR (RPL [All Fields]) OR (("aborted fetus"[MeSH Terms] OR ("aborted"[All Fields] AND "fetus"[All Fields]) OR "aborted fetus"[All Fields] OR "abortus"[All Fields]) AND "provocatus"[All Fields]) OR "miscarriage"[All Fields])) AND ((clinicalstudy[Filter] OR clinicaltrial[Filter] OR clinicaltrialphaseiii[Filter] OR clinicaltrialphaseiv[Filter] OR controlledclinicaltrial[Filter] OR meta-analysis[Filter] OR multicenterstudy[Filter] OR randomizedcontrolledtrial[Filter] OR systematicreview[Filter]) AND (humans[Filter]) AND (female[Filter]) AND (afrikaans[Filter] OR dutch[Filter] OR english[Filter]) AND (2000:2021[pdat]))

(("polycystic"[All Fields] AND "ovarian"[All Fields] AND "syndrome"[All Fields]) OR ("polycystic"[All Fields] AND "ovary"[All Fields] AND "syndrome"[All Fields]) OR "polycystic ovary syndrome"[All Fields] OR "PCOS"[All Fields]) AND (("recurrent"[All Fields] AND "pregnancy"[All Fields] AND "loss"[All Fields]) OR "recurrent miscarriage"[All Fields] OR "RPL"[All Fields] OR (("aborted fetus"[MeSH Terms] OR ("aborted"[All Fields] AND "fetus"[All Fields]) OR "aborted fetus"[All Fields] OR "abortus"[All Fields]) AND "provocatus"[All Fields]) OR "miscarriage"[All Fields]) AND (("clinical study"[Publication Type] OR "clinical trial"[Publication Type] OR "clinical trial, phase iii"[Publication Type] OR "clinical trial, phase iv"[Publication Type] OR "controlled clinical trial"[Publication Type] OR "meta analysis"[Publication Type] OR "multicenter study"[Publication Type] OR "randomized controlled trial"[Publication Type] OR "systematic review"[Filter]) AND "humans"[MeSH Terms] AND "female"[MeSH Terms] AND ("afrikaans"[Language] OR "dutch"[Language] OR "english"[Language]) AND 2000/01/01:2021/12/31[Date - Publication])

Scopus

## (TITLE-ABS-KEY ( ( "polycystic ovary syndrome"  OR  "polycystic ovarian syndrome"  OR  pcos )  AND  ( "recurrent pregnancy loss"  OR  "recurrent miscarriage"  OR  rpl ) )  AND  ( LIMIT-TO ( SRCTYPE ,  "j" ) )  AND  ( LIMIT-TO ( PUBSTAGE ,  "final" ) )  AND  ( LIMIT-TO ( DOCTYPE ,  "ar" ) )  AND  ( LIMIT-TO ( LANGUAGE ,  "English" ) )  AND  ( LIMIT-TO ( PUBYEAR ,  2021 )  OR  LIMIT-TO ( PUBYEAR ,  2020 )  OR  LIMIT-TO ( PUBYEAR ,  2019 )  OR  LIMIT-TO ( PUBYEAR ,  2018 )  OR  LIMIT-TO ( PUBYEAR ,  2017 )  OR  LIMIT-TO ( PUBYEAR ,  2016 )  OR  LIMIT-TO ( PUBYEAR ,  2015 )  OR  LIMIT-TO ( PUBYEAR ,  2014 )  OR  LIMIT-TO ( PUBYEAR ,  2013 )  OR  LIMIT-TO ( PUBYEAR ,  2012 )  OR  LIMIT-TO ( PUBYEAR ,  2011 )  OR  LIMIT-TO ( PUBYEAR ,  2010 )  OR  LIMIT-TO ( PUBYEAR ,  2009 )  OR  LIMIT-TO ( PUBYEAR ,  2008 )  OR  LIMIT-TO ( PUBYEAR ,  2007 )  OR  LIMIT-TO ( PUBYEAR ,  2006 )  OR  LIMIT-TO ( PUBYEAR ,  2004 )  OR  LIMIT-TO ( PUBYEAR ,  2003 )  OR  LIMIT-TO ( PUBYEAR ,  2002 )  OR  LIMIT-TO ( PUBYEAR ,  2001 )  OR  LIMIT-TO ( PUBYEAR ,  2000 ) )

Science direct:

Search: Title, abstract: polycystic ovary syndrome, polycystic ovarian syndrome, pcos, recurrent pregnancy loss, recurrent miscarriage, RPL, aborted fetus, abortus provocatus years 2000-2021

Search refined by article type: ‘review articles’, ‘research articles’, ‘other’

Cochrane library:

Search using Advanced Search manager. #1 MeSH descriptor: [Polycystic Ovary Syndrome] explode all trees, #2 MeSH descriptor: [Abortion, Habitual] explode all trees. #3 #1 AND #2. Filter for year: 2000-2021

EBSCO host:

Select all databases

Search using Advanced Search manager: PCOS OR “polycystic ovarian syndrome” OR “polycystic ovary syndrome” [in subject terms] AND “recurrent pregnancy loss” OR RPL OR “recurrent miscarriage” OR “aborted fetus” OR “abortus provocatus” [in subject terms]. Years 2000-2021, language: English

# Supplementary table: characteristics of analyzed studies

|  | Study design | Country of participant recruitment | Study objective (main) | Study population | Number of previous miscarriages in study population | Definition of PCOS |  |
| --- | --- | --- | --- | --- | --- | --- | --- |
| Khomani et. al. (2019) | Systematic Review | - | Prevalence of pregnancy- & delivery complications in women with PCOS | - | Not specified | Any criteria (NIH, AES, ESHRE/ASRM) |  |
| Morley et. al. (2017) | Systematic Review | - | Insulin-sensitizing drugs | - | Not specified | Rotterdam criteria |  |
| Ma et. al. (2021) | Systematic Review | - | Association between hyperandrogenism and adverse pregnancy outcomes in PCOS patients | - | Not specified | Rotterdam criteria |  |
| Boomsma et. al. (2006) | Meta-analysis | - | Pregnancy outcomes in PCOS | - | Not specified | Rotterdam criteria |  |
| Morin-Papunen et. al. (2012) | Randomized Controlled Trial | Finland | Metformin | PCOS + Metformin (n=160)  PCOS + placebo (n=160) | Not specified | Rotterdam criteria |  |
| Løvvik et. al. (2019) | Randomized Controlled Trial | Norway, Sweden, Iceland | Metformin | PCOS + Metformin (n=230)  PCOS + placebo (n=240) | 0, 1, 2, ≥3 | Rotterdam criteria |  |
| Rai et. al. (2000) | Prospective cohort study | United Kingdom | PCO in RPL | RPL (n = 2199)  Pregnant, normal ovaries (n=253)  Pregnant, PCO (n= 233) | ≥3 | - |  |
| Chakraborty et. al. (2013) | Prospective cohort study | India | Aspirin & Aspirin-LMWH | PCOS (n=147)  Non-PCOS (n=189) | ≥2, ≥3 | Rotterdam criteria |  |
| Zolghadri et. al. (2007) | Prospective cohort study | Iran | Metformin | RPL (n=164)  Controls (n=74) | ≥3 | Major criteria: chronic oligomenorrhea, clinical and biochemical hyperandrogenism, exclusion of other etiologies. Other criteria: polycystic ovaries on imaging |  |
| De Leo (2006) | Prospective cohort study | Italy | Metformin | PCOS + Metformin (n=98)  Control + Metformin (n=110) | Not specified | Rotterdam criteria |  |
| Khattab et. al. (2009) | Prospective cohort study | Egypt | Metformin | PCOS + Metformin (n=120)  PCOS, no Metformin (n=80) | Not specified | Rotterdam criteria |  |
| Szafarowska et. al. (2018) | Prospective cohort study (preliminary report) | Poland | AMH | PCOS+RPL (n=37)  PCOS-infertile (n=7)  Healthy controls (n=20) | ≥2 | Rotterdam criteria |  |
| Kazerooni et. al. (2013) | Case-control study | Iran | Trombophilia & RPL in PCOS | RPL + PCOS (n=60)  PCOS (n=60)  RPL (n=60)  Healthy controls (n=60) | ≥3 | Rotterdam criteria |  |
| Kargasheh et. al. (2001) | Case-control study | Iran | Leptin & sOB-R | PCOS (n=324)  PCOS-infertile (n=199)  PCOS-RPL (n=125)  Healthy controls (n=144) | Not specified | Rotterdam criteria |  |
| Fouani et. al. (2020) | Case-control study | Iran | Metrnl | PCOS-RPL (n=60)  PCOS-infertile (n=60)  Controls (n=60) | ≥2 | Rotterdam criteria |  |
| Rogenhofer et. al. (2013) | Case-control study | Germany | M2 Haplotype of ANXA5 | PCOS, no previous pregnancies (n=27)  PCOS, primary RPL (n=45)  PCOS, secondary RPL (n=28)  Fertile controls (n=500) | ≥2 | Rotterdam criteria |  |
| Alkhuriji et. al. (2020) | Case-control study | Saudi Arabia | IL-1- β, IL-6, TNF- α & TGF β1 polymorphisms | RPL (n=70)  RPL+PCOS (n=70)  Healthy controls (n=140) | ≥3 | Rotterdam criteria |  |
| Nawaz et. al. (2009) | Case-control study | Pakistan | Metformin | PCOS + Metformin (n=119)  PCOS, no Metformin (n=78) | Not specified | Rotterdam criteria |  |
| Moini et. al. (2012) | Case-control study | Iran | Thrombophilic disorders | PCOS-RPL (n=92)  RPL (n=92) | ≥3 | Rotterdam criteria |  |
| Idali et. al. (2012) | Case-control study | Iran | PAI-I & MTHFR gene mutations | RPL+PCOS (n=38)  RPL + ovarian PCO (n=33)  RPL (n=106)  Healthy controls (n=100) | ≥3 | Rotterdam criteria |  |
| Szafarowska et. al. (2016) | Case-control study | Poland | MTHFR gene mutations | PCOS + RPL (n=63)  PCOS-infertile (n=13)  Non-PCOS +RPL (n=40)  Non-PCOS -infertile (n=16) | ≥2 | Rotterdam criteria |  |
| Sadeghi et. al. (2020) | Case-control study | Iran | CTRP-6 & adiponectin | PCOS-RPL (n=60)  PCOS-infertile (n=60)  Non-PCOS (n=60) | ≥2 | Rotterdam criteria |  |
| Glueck et. al. (1999) | Case-control study | USA | PAI-Fx in PCOS | PCOS (n=108)  PCOS, ≥1 pregnancy (n=41) | ≥1 | ≥2 major criteria; polycystic ovaries on imaging, chronic oligomenorrhoea, clinical hyperandrogenism. Other criteria; LH to FSH ratio >1,5 in amenorrheic state, morbid obesity, acanthosis nigrans |  |
| Asanidze et. al. (2019) | Case-control study | Georgia | Homocysteine, AMH, insuline resistance | PCOS + RPL (n=50)  PCOS + live births (n=30) | Not specified | Rotterdam criteria |  |
| Glueck et. al. (2002) | Case-control study | USA | Metformin | PCOS (n=72) | Not specified | Chronic oligomenorrhoea + clinical hyperandrogenism or biochemical hyperandrogenism. |  |
| Glueck et. al. (2003) | Case-control study | USA | PAI-I activity and Factor V Leiden mutation | PCOS + RPL (n=33)  RPL (n=16)  Non-PCOS, non-RPL (116) | ≥3 | 1990 National Institues of Health criteria: oligo-amenorrhoea, biochemical or clinical evidence of hyperandrogenism, exclusion of other disorders. |  |
| Glueck et. al. (2006) | Case-control study | USA | PAI-I activity level | PCOS (n=967)  Non-PCOS (n=126) | Not specified | Rotterdam criteria |  |
| Glueck et. al. (2013) | Case-control study | USA | Metformin + diet | PCOS (76)  Non-PCOS (n=156) | Not specified | Rotterdam criteria |  |
| Tarkun et. al (2004) | Case-control study | Turkey | Insulin resistance and CRP | PCOS (n=37)  Non-PCOS (n=25) | Not specified | Rotterdam criteria |  |
| Chakraborty et. al. (2013) | Case-control study | India | Hyperhomocysteinemia & insulin resistance | PCOS (n=126)  Non-PCOS (n=117) | ≥2 | Rotterdam criteria |  |
| Rees et. al (2016) | Case-control study | United Kingdom | Effect of PCOS on fertility, pregnancy, and neonatal outcomes | PCOS (n=9680)  Non-PCOS (n=18136) | Not specified | ICD-10 |  |
| Jacubowicz (2002) | Case-control study | Venezuela | Metformin | PCOS + Metformin (n=65)  PCOS, no Metformin (n=31) | Not specified | Oligomenorrhoea, hyperandrogenism, polycystic ovaries on imaging |  |
| Wang et. al (2001) | Case-control study | Australia | Effect of PCOS on risk of spontaneous abortion | PCOS (n=373)  Non-PCOS (n=645) | ≥3 | Serum testosterone >2,5 nmol/l or elevated androstenedione with a low SHBG concentration + polycystic ovaries on imaging |  |
| Okon et. al. (1998) | Case-control study | United Kingdom | Serum androgen levels | RPL (n=33)  RPL + PCOD and/ or early follicular phase LH >10IU/L (n=10) | ≥3 | PCOD features on imaging or early follicular phase LH >10IU/L |  |
| Ramidi et. al. (2009) | Case series | USA | Metformin-enoxaparin | PCOS (n=21)  Coagulation disorders (n=7)  Healthy controls (n=51) | 1, 2, ≥ 3 | Rotterdam criteria |  |
| Glueck et. al. (2004) | Case series | USA | Metformin-enoxaparin | PCOS (n=24)  Healthy controls (n=137) | 1, 2, ≥3 | Chronic oligomenorrhoea, clinical hyper-androgenism and/ or biochemical hyper-androgenism |  |
| Glueck et. al. (2001) | Case series, pilot study | USA | Metformin, | PCOS (n=22)  Healthy controls for comparison of PAI activity (n=23)  Healthy controls for comparison of 4G polymorphism of PAI-I gene (n=109) | Not specified | Major criteria: chronic oligo-amenorrhoea, clinical and biochemical hyperandrogenism, exclusion of other etiologies. Other criteria; polycystic ovaries on imaging, acanthosis nigrans, LH/FSH ratio ≥2. |  |
